# Effects of Ginkgo leaf tablets on the pharmacokinetics of losartan and its metabolite EXP3174 in rats and its mechanism

**DOI:** 10.1080/13880209.2018.1481107

**Published:** 2018-06-28

**Authors:** Baiping Dong, Suowei Yuan, Jinsheng Hu, Yanzhen Yan

**Affiliations:** Department of Neurology, Caoxian People’s Hospital, Heze, Shandong Province, China

**Keywords:** Herb–drug interaction, metabolism, CYP3A4

## Abstract

**Context:** Ginkgo leaf tablets (GLTs) and losartan are often simultaneously used for the treatment of hypertension in Chinese clinics. However, the herb–drug interaction between GLT and losartan is still unknown.

**Objective:** This study investigates the effects of GLT on the pharmacokinetics of losartan and its metabolite EXP3174 in rats and its potential mechanism.

**Materials and methods:** The pharmacokinetic profiles of losartan and EXP3174 of orally administered losartan (10 mg/kg) with or without GLT pretreatment (80 mg/kg/day for 10 days) in Sprague–Dawley rats were determined. *In vitro*, the effects of GLT on the metabolic stability of losartan were investigated with rat liver microsomes.

**Results:** The *C*_max_ (1.22 ± 0.25 *vs* 1.85 ± 0.37 μg/mL) and the *AUC*_(0–_*_t_*_)_ (6.99 ± 1.05 *vs* 11.94 ± 1.79 mg·h/L) of losartan increased significantly (*p* < 0.05) with GLT pretreatment, while the *C*_max_ (1.05 ± 0.19 *vs* 0.72 ± 0.12 μg/mL) of EXP3174 decreased significantly (*p* < 0.05) compared to the control. The *t*_1/2_ of losartan was prolonged significantly from 3.94 ± 0.62 to 4.75 ± 0.52 h (*p* < 0.05). The metabolic stability of losartan was increased from 37.4 min to 59.6 min with GLT pretreatment.

**Discussion and conclusions:** The results indicate that GLT might increase the plasma concentration of losartan and decrease the concentration of EXP3174 through inhibiting the metabolism of losartan.

## Introduction

Losartan is the first nonpeptide angiotensin II receptor blocker used in hypertension and diabetic nephropathy (Klishadi et al. 2015). Losartan can be absorbed quickly and transformed into its active metabolite EXP3174 after oral administration, and the clinical efficacy of EXP3174 is about 10-fold more potent than its parent drug (Rincon et al. 2015). Thus, the clinical hypotensive activity is predominantly mediated by the active metabolite EXP3174, although losartan itself exhibits good efficacy (Varshney et al. 2013). Because of its good anti-hypertension effect, losartan has been one of the anti-hypertension drug most frequently used for the prevention and control of hypertension in the clinic (Yasar et al. 2008; Yang et al. 2011). Losartan is mainly metabolized by cytochrome P450 enzymes CYP3A4 and CYP2C9, and therefore, modulation of CYP3A4 or CYP2C9 activities may cause significant changes in the pharmacokinetic profiles of losartan and its active metabolite EXP3174 (Li et al. 2009; Wang et al. 2016; Hu et al. 2017).

Ginkgo leaf tablet (GLT) is an effective traditional Chinese multi-herbal formula which is widely used in treating ischemic cerebrovascular disease in the clinic (Lin et al. 2003; Chung et al. 2006; Yang et al. 2017). The main components of GLT are ginkgo flavone glycosides, ginkgolides and bilobalides (Liu et al. 2009; Guan et al. 2014; Rao et al. 2014). Losartan and GLT are often simultaneously used for the treatment of hypertension in Chinese clinics. However, many herb–drug interactions resulting from concurrent use of herbal drugs with over-the-counter drugs may cause adverse reactions such as toxicity and treatment failure, and the herb–drug interaction between GLT and losartan are still unknown. Therefore, it is essential to investigate the effects of GLT on the pharmacokinetics of losartan and its potential mechanism.

This study investigates the effects of GLT on the pharmacokinetics of losartan and clarifies its main potential mechanism using rat liver microsome incubation systems.

## Materials and methods

### Chemicals and reagents

Standards of losartan (purity >98%) was purchased from the National Institute for the Control of Pharmaceutical and Biological Products (Beijing, China). Losartan carboxylic acid (EXP3174) was purchased from Toronto Research Chemicals Inc., Canada. GLTs were purchased from Sichuan Kelun Pharmaceutical Co., LTD. β-Nicotinamide adenine dinucleotide phosphate (NADP) and lucifer yellow were provided by Sigma (St. Louis, MO). Rat liver microsomes were purchased from BD (Woburn, MA). Acetonitrile and methanol were purchased from Fisher Scientific (Fair Lawn, NJ). Formic acid was purchased from Anaqua Chemicals Supply Inc. Limited (Houston, TX). Ultrapure water was prepared with a Milli-Q water purification system (Millipore, Billerica, MA). All other chemicals were of analytical grade or better.

### Instrumentation and conditions

The analysis was performed on an Agilent Series 1100 HPLC system and Agilent G1946 single quadrupole mass spectrometer (Palo Alto, CA) as previously reported (Li et al. 2016). The sample was separated on Waters Xbridge C18 column (100 × 3.0 mm, i.d.; 3.5 mm, USA) and eluted with an isocratic mobile phase: solvent A (H_2_O–HCOOH, 100:0.1, v/v) and solvent B (CH_3_OH) (55:45, v/v). The total analysis time was 8 min. The column temperature was 25 °C at a flow rate of 0.8 mL/min and injection volume of 5 μL, and the split ratio was 1:1.

The mass conditions were optimized as follows: capillary 4000 V, nebulizer pressure 40 psig, drying gas flowing rate 10 L/min, gas temperature 350 °C and fragmentor 80 ev. Select-ion-monitoring (SIM) in the positive ion mode was used. The ions of losartan, EXP3174, and irbesartan were recorded as *m*/*z* 423.1, *m*/*z* 437.1, and *m*/*z* 429.2 for the quantification, respectively.

### Animal experiments

Male Sprague–Dawley rats weighing 250 ± 20 g were provided by the experimental animal center of the Taishan Medical University (Taishan, China). The rats were maintained in an air-conditioned animal quarter at 22 ± 2 °C and 50 ± 10% relative humidity. Water and food (laboratory rodent chow, Shanghai, China) were allowed ad libitum. The animals were acclimatized to the facilities for 5 days, and then fasted with free access to water for 12 h prior to each experiment. All experimental procedures and protocols were reviewed and approved by the Animal Care and Use Committee of Taishan Medical University and were in accordance with the National Institutes of Health guidelines regarding the principles of animal care.

### *In vivo* pharmacokinetic study

Twelve rats were equally randomized to two groups (six rats in each group), including the losartan group (A) and the losartan + GLT group (B). Group B was pretreated with GLT solutions at a dose of 80 mg/kg/day for 10 days before the administration of losartan, and then losartan were orally administered to rats by gavage at a dose of 10 mg/kg. Blood samples (0.20 mL) were collected into a heparinized tube via the *oculi chorioideae* vein before drug administration and at 0.083, 0.25, 0.5, 1, 2, 3, 4, 6, 8, 12 and 24 h after drug administration. The blood samples were centrifuged at 3500 rpm for 5 min. The plasma samples that were obtained were stored at −40 °C until analysis.

### Effects of the main components in GLT on the metabolism of losartan in rat liver microsomes

The effects of GLT on the metabolic stability of losartan were investigated using rat liver microsome incubation systems. The assay conditions and reaction mixtures were similar as reported previously (Li et al. 2016). In brief, 10 μL rat liver microsomes (20 mg/mL), 4 μL losartan solution (100 μM, final concentration of 1 μM) and 366 μL PBS buffer were added to the centrifuge tubes on ice. The reaction mixture was incubated at 37 °C for 5 min and then NADPH-generating system (15 μL) was added. The effects of the above components on the metabolic stability of losartan were investigated by adding 4 μL GLT solution (350 μg/mL) to rat liver microsomes and preincubating them for 30 min at 37 °C, followed by the addition of losartan. Aliquots of 30 μL were collected from reaction volumes at 0, 1, 3, 5, 15, 30 and 60 min and 60 μL ice-cold acetonitrile containing IS was added to terminate the reaction, and then the concentration of losartan was determined using LC–MS. The half-life (*t*_1/2_) *in vitro* was obtained using equation: *t*_1/2_ = 0.693/k.

### Data analysis

The pharmacokinetic parameters were calculated using the DAS 3.0 pharmacokinetic software (Chinese Pharmacological Association). The differences between the mean values were analyzed for significance using a one-way analysis of variance (ANOVA). Values of *p <* 0.05 were considered to be statistically significant.

## Results

### *In vivo* pharmacokinetic study

**Figure 1.**
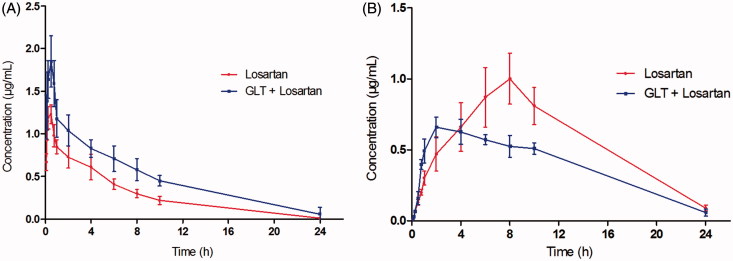
The mean concentration-time curves in rat plasma after oral administration of losartan or both GLT and losartan. (A) losartan; (B) EXP3174.

The pharmacokinetic parameters of losartan and EXP3174 were calculated using the noncompartmental method with DAS 3.0 pharmacokinetic software, and the pharmacokinetic parameters are shown in Table 1. The mean plasma concentration–time curves of losartan and EXP3174 are shown in Figure 1.

**Table 1.** Pharmacokinetic parameters of losartan and EXP3174 in male Sprague–Dawley rats following oral administration of losartan alone (Group A) or both losartan and GLT (Group B).

Parameter	Losartan		EXP3174	
	Group A	Group B	Group A	Group B
*T*_max_ (h)	0.39 ± 0.08	0.51 ± 0.10*	7.98 ± 1.02	3.34 ± 0.63*
*C*_max_ (mg L^−1^)	1.22 ± 0.25	1.85 ± 0.37*	1.05 ± 0.19	0.72 ± 0.12*
*t*_1/2_ (h)	3.94 ± 0.62	4.75 ± 0.52*	4.42 ± 0.35	4.99 ± 0.51
AUC_(0–t)_ (mg h L^−1^)	6.99 ± 1.05	11.94 ± 1.79*	13.12 ± 2.52	7.28 ± 1.07*
Oral CL (L h^−1^ kg^−1^)	1.43 ± 0.29	0.82 ± 0.21*	0.75 ± 0.14	1.29 ± 0.25*

*p < 0.05 indicates significant differences from the control.

As shown in Table 1, the parameters *C*_max_ and AUC_(0–_*_t_*_)_ for losartan in the losartan and GLT (B) group were larger than those of losartan-only group (A), and the difference was significant (*p* < 0.05); However, *C*_max_ and AUC_(0–t)_ for EXP3174 in the losartan and GLT (B) group were smaller than those of losartan-only group (A), and the difference was also significant (*p* < 0.05). The results indicated that the plasma concentration of losartan increased in losartan and GLT group compared with the single losartan group, and however, the plasma concentration of EXP3174 decreased. The *t*_1/2_ of losartan was prolonged significantly with the pretreatment of GLT, and the oral clearance rate decreased significantly (*p* < 0.05). The results showed that the metabolism clearance of losartan was inhibited by GLT.

### Effects of GLT on the metabolic stability of losartan in rat liver microsome incubation systems

As we know, the metabolism of losartan was mainly mediated by CYP450 enzyme, and therefore, in this research, the effects of GLT on the metabolic rate of losartan were investigated. The results showed that the metabolic stability of losartan was 32.5 min, while the metabolic rate was prolonged (46.8 min) in the presence of GLT. The results indicated that GLT could inhibit the metabolic stability of losartan.

## Discussion

This is the first study to investigate the effects of GLT on the pharmacokinetics of losartan and its metabolite EXP3174 in rats, and the results indicated that GLT could increase the systemic exposure of losartan and while decrease the systemic exposure of EXP3174 in rats. The rat liver microsome incubation experiments revealed that GLT might affect the pharmacokinetics profiles of losartan and EXP3174 through inhibiting its metabolism in rat liver.

It is now well known that a drug can inhibit the metabolic stability of another drug, leading to a higher than intended plasma level (Wrighton and Stevens 1992; Zhang et al. 2007; Ye et al. 2011). The major clinical consequence of inhibitory drug–drug interactions is undesired drug toxicity (Gouws et al. 2012; Qi et al. 2013). For example, the antifungal ketoconazole, a potent inhibitor of CYP3A4, causes drug–drug interactions with drugs that are substrates of CYP3A4 (Xiaoyang et al. 2015). Losartan was primarily metabolized by CYP3A4 and CYP2C19 in liver, and therefore, herbs or drugs that could affect the activity of CYP3A4 or CYP2C19 might influence the pharmacokinetics profiles of losartan when they are co-administered. Some research articles have also indicated that the pharmacokinetic profiles of losartan was affected by co-administered herbs or drugs (Spanakis et al. 2013; Yuan et al. 2013; Li et al. 2016; Wang et al. 2016).

Previous studies have also reported that the GLT could affect the pharmacokinetic profiles of amlodipine, cilostazol, fexofenadine and clopidogrel when they are co-administered with GLT (Deng et al. 2016; Wang et al. 2016; Turkanovic et al. 2017). The main components in GLT are ginkgolides A, ginkgolides B, bilobalide, quercetin, and kaempferol, and several studies have indicated that the main components in could inhibit the activity of CYP3A4 enzymes (Etheridge et al. 2007; Zadoyan et al. 2012; Palle and Neerati 2017).

Therefore, we think that GLT might affect the pharmacokinetic profiles of losartan and EXP3174 through inhibiting the metabolism of losartan. The present study reminds us that the herb–drug interaction between GLT and losartan might happen, and further evaluation in clinical studies is also necessary.
